# Flexural Fatigue Properties of Ultra-High Performance Engineered Cementitious Composites (UHP-ECC) Reinforced by Polymer Fibers

**DOI:** 10.3390/polym10080892

**Published:** 2018-08-09

**Authors:** Lili Sui, Qianli Zhong, Kequan Yu, Feng Xing, Pengda Li, Yingwu Zhou

**Affiliations:** 1 Guangdong Provincial Key Laboratory of Durability for Marine Civil Engineering, Shenzhen University, Shenzhen 518060, China; suill8969@163.com (L.S.); 2160150404@email.szu.edu.cn (Q.Z.); xingf@szu.edu.cn (F.X.); pdli.peter@szu.edu.cn (P.L.); 2 Department of Civil and Environmental Engineering, The Hong Kong Polytechnic University, Hung Hom, HongKong, China; zjzjykq@163.com

**Keywords:** ultra-high performance engineered cementitious composites, flexural fatigue, stress level, fatigue life, fiber morphology

## Abstract

In recent years, the application of engineered cementitious composites (ECCs) in structures subjected to cyclic fatigue loading, such as highway bridges, has gained widespread attention. However, most existing ECCs do not have sufficient strength and ductility, which limits their applications, especially in highway bridge structures under high-stress. In this work, an ultra-high performance engineered cementitious composite (UHP-ECC) was configured, which had a compressive strength of approximately 120 MPa, a tensile strength of up to 12 MPa, and a tensile strain capacity of more than 8%. This paper presents a study of the fatigue performance of UHP-ECC at four different fatigue stress levels through the four-point bending test. The mid-span deflection of the specimen was monitored along with the crack opening displacement (COD) of the pure bending section at the bottom of the specimen, and the crack width. In addition, the dissipated energy was calculated at various stress levels. The progressive formation of cracks under static loading was monitored using the digital image correlation (DIC) technique. The fibers at the fractured surface of the specimens were observed and analyzed by environmental scanning electron microscopy, and the morphology of the fibers was obtained at different fatigue stress levels. Eventually, the fatigue life under different stress levels was obtained, and the relationship between the fatigue life and the stress level was established.

## 1. Introduction

The engineered cementitious composite (ECC) is fiber reinforced and designed using micromechanical principles, which shows high ductility under uniaxial tensile and shear stress [1]. The ECC enters the plastic deformation stage after the initial cracks are generated. The strain-hardening process is accompanied by the continuous generation and development of micro-cracks. The ultimate tensile strain capacity of the conventional polyvinyl alcohol fiber ECC (PVA-ECC) exceeds 3%, which is 200 times that of concrete [2]. The crack width of ECCs falls within a reasonable range due to the constraint of the maximum bridging stress. Even during the stage where saturated multiple cracks are formed, the maximum crack width of PVA-ECCs can be maintained below 100 μm [3].

**Figure 1:**
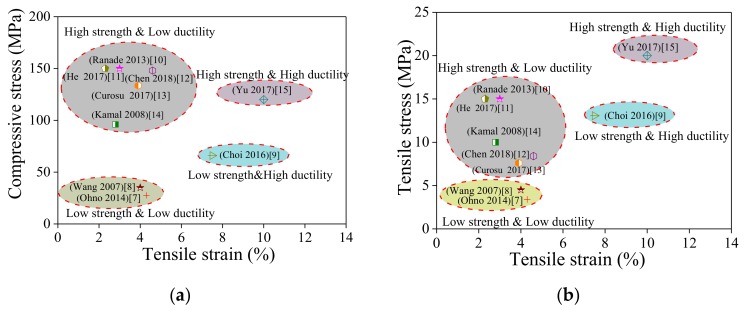
A summary of ECC strength and ductility in existing literature: (**a**) Compressive stress-tensile strain; (**b**) Tensile stress-tensile strain.

When considering highway bridge pavement and bridge expansion joints subject to fatigue loading, climate change, and vehicle overloading, concrete is easily damaged because of its inherent brittleness, resulting in frequent repairs and high economic and social costs [4]. The crack control ability and strain capacity of ECCs meet the deformation requirements of bridge connecting plates, showing a great potential in constructing continuous bridge structures [5,6]. The developmental history of ECCs reveals that the ECC is mainly divided into four types according to strength and ductility, as shown in Figure 1, including low-strength/low-ductility ECCs [7,8], low-strength/high-ductility ECCs [9], high-strength/low-ductility ECCs [10,11,12,13,14], and high-strength/high-ductility ECCs [15]. Many scholars have mainly conducted studies on flexural fatigue performance [16,17,18,19,20,21], uniaxial tensile fatigue [22,23,24,25], and fiber-matrix interfacial adhesion [26,27,28] pertaining to the first type of ECCs, i.e., the low-strength/low-ductility ECC. The fatigue properties of other types of ECCs have not been studied. Suthiwarapirak et al. [17] compared the flexural fatigue life of PVA-ECC, polyethylene fiber-ECC (PE-ECC), steel fiber reinforced cement (SFRC), and polymer cement mortar (PCM). The results show that the fatigue life of PCM is far less than that of the other three types of fiber concrete. The fatigue performance of PE-ECC and SFRC is similar. Compared with PE-ECC, the fatigue life of PVA-ECC improves at high stress levels, but decreases at low stress levels. Liu et al. [19,20] established a fatigue damage model under constant-amplitude cyclic loading and derived an equation to predict flexural fatigue strength. Mohamed et al. [21] compared ECC mixtures deflection capacity under static and fatigue flexural loading using local crushed sand and supplementary cementing material. Researchers have also conducted in-depth studies on the direct tensile fatigue of PVA-ECCs under different loading regimes [25] and different scales as well as established corresponding micromechanical models [26,27,28].

The compressive strength of conventional PVA-ECCs generally does not exceed 50 MPa due to the mechanical properties of PVA fibers and the adhesion between the PVA fibers and the matrix [29,30]. In addition, due to the lack of coarse aggregates in ECCs, the elastic modulus of ECCs is less than that of concrete with the same compressive strength. PE-ECCs with higher mechanical strength, better ductility, and higher tensile strength were designed by using ultra-high molecular weight polyethylene (PE) fibers, which are stronger than PVA fibers and have a higher elastic modulus. The tensile strength of PE-ECCs is up to 20 MPa, and the tensile strain capacity reaches more than 8% [31], which is two orders of magnitude higher than that of concrete. The maximum compressive strength can reach 120 MPa [32]. Due to its excellent mechanical properties, this new ECC is referred to as ultra-high performance engineered cementitious composites (UHP-ECCs). Although many researchers have conducted in-depth studies on the fatigue properties of PVA-ECCs, studies on the fatigue properties of PE-ECCs are relatively lacking, and the fatigue properties of UHP-ECCs have not been investigated. Therefore, this paper mainly focuses on the fatigue properties of UHP-ECCs.

This paper aims to determine the fatigue life of UHP-ECCs at different stress levels by performing a four-point bending fatigue test. During the test, the mid-span deflection of the specimen, the crack opening displacement (COD) of the pure bending section at the bottom of the specimen, and the crack width were monitored. The digital image correlation (DIC) technique was used to observe the progress of the cracks of specimens under static loading. Scanning electron microscopy was employed to observe and analyze the fiber morphology at different stress-level failures. The equation for predicting the fatigue life of UHP-ECCs was established, and the cumulative fatigue damage rules and failure modes of UHP-ECCs under different cyclic loading were established.

## 2. Experimental Program

### 2.1. Raw Materials and Composition

**Figure 2:**
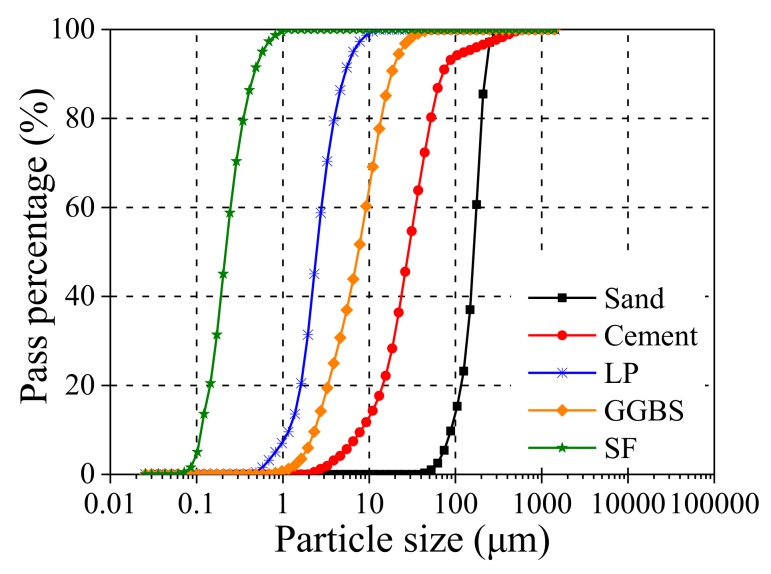
Gradation curves of cementitious materials and silica sand were determined from particle size analysis.

UHP-ECC consists of five components, i.e., cementitious material, water reducer, aggregate, water, and fiber (Table 1). The cementitious material includes ordinary Portland cement (OPC) 52.5R, ground granulated blast furnace slag (GGBS), limestone powder (LP), and silica fume (SF). GGBS and SF promote the formation of secondary hydration products. In this study, fine LP was mainly used as a filler and utilized to partially replace the cement, and it is also believed to accelerate the C_3_S hydration, and to react with C_3_A in the presence of water to form calcium carboaluminates (C_3_A·CaCO_3_·11H_2_O) [33,34]. The chemical composition of cementitious materials (OPC, SF, GGBS, and LP) is shown in Table 2, which shows the physical, chemical and mechanical properties of cement based on the Chinese cement code (GB175-2007) [35]. The particle size distribution is shown in Figure 2, which shows limestone powder with a particle size between that of silica fume and mineral powder, which sets limestone powder apart as an effective filler. The selected cementitious material not only promotes the formation of the C-S-H gel, but also improves the homogeneity of the mixture, thereby increasing both compressive strength and tensile strength. Polycarboxylate-based high-range water reducer (HRWR) was used to ensure the fluidity of UHP-ECCs. The aggregate was fine quartz sand with an average particle size of 100 μm and a maximum particle size of 181 μm. The volume fraction of the PE fibers was 2%. The major physical properties of the PE fibers are shown in Table 3.

### 2.2. Specimen Design

**Figure 3:**
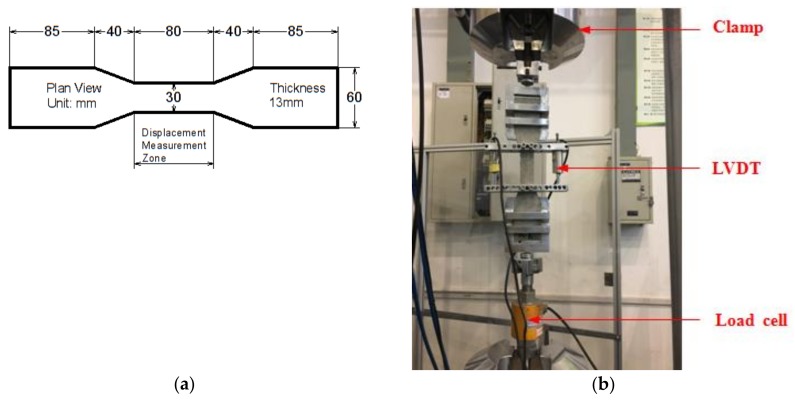
Set-up of tensile test: (**a**) dimension of dog bone specimen; and (**b**) setup.

**Figure 4:**
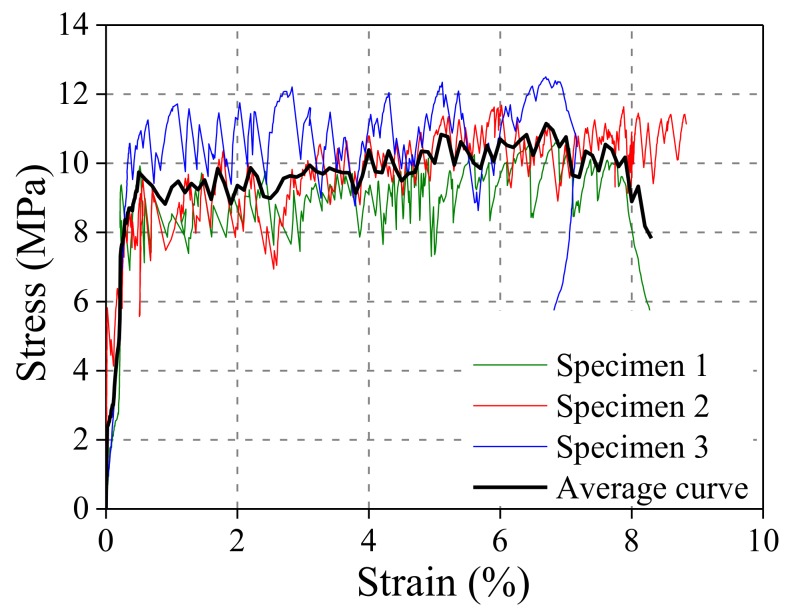
Tensile stress-strain curves of UHP-ECC.

**Figure 5:**
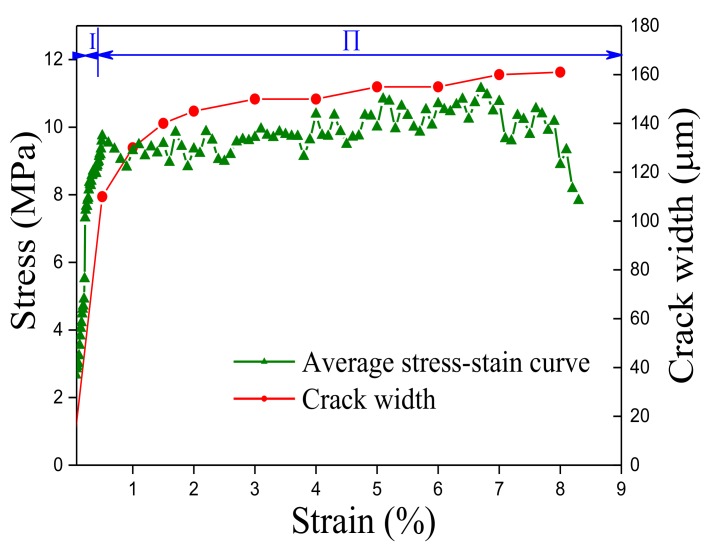
Development of steady state crack opening in UHP-ECC with increasing strain.

A tensile test was first conducted to obtain the tensile mechanical properties of ECCs. Three dog-bone shaped specimens were tested. The dimensions of the dog-bone shaped specimen and the loading device are shown in Figure 3. In the gauge segment, two linear variable differential transformers (LVDTs) were used to measure the tensile deformation for the calculation of real-time strain. The tensile stress-strain curve is shown in Figure 4. In the uniaxial tensile test, the crack widths were measured at the specified tensile displacement using a crack-width gauge, and the average crack width was calculated to obtain a relationship between the average crack width and the strain, as shown in Figure 5. Figure 5 shows that two significant stages can be distinguished where Stage I is defined as the crack width’s rapidly increasing stage, and Stage II is the crack width stable development stage. Due to the presence of fiber bridging, the crack width of UHP-ECC was controlled under 160 μm.

In this paper, a four-point bending beam was used for the fatigue test. A total of 15 beams’ specimens were prepared for this study. The specimen dimension was 100 mm × 100 mm × 400 mm, and the specimens were labeled in the format of U-*S*-*n*, where, U refers to UHP-ECC, *S* stands for the fatigue stress level, i.e., the ratio of the applied maximum fatigue stress σ_max_ to the average flexural strength *f*_a_, which includes four levels of 0.5, 0.65, 0.8, and 0.9, and *n* is expressed as the serial number of the specimens in each group. Specifically, *S* = 1.0 means the specimen was statically loaded to failure, i.e., the static non-cycle test. Six standard cubes of 100 mm × 100 mm × 100 mm were also manufactured at the same time for each batch of specimens to determine the compressive mechanical properties of the UHP-ECC. The specimens were demolded on the second day of casting and placed in a standard curing room under the conditions of 95% relative humidity (RH) and 20 ± 2 °C temperature. All specimens were cured for 90 days to eliminate the curing effect on the mechanical properties. The average compressive strength of the UHP-ECC specimens at 28 days and 90 days was 102.8 MPa and 120.7 MPa, respectively. The standard deviation of the UHP-ECC specimens at 28 days and 90 days was 1.1 and 0.75, respectively.

### 2.3. Load Setup and Measurement

**Figure 6:**
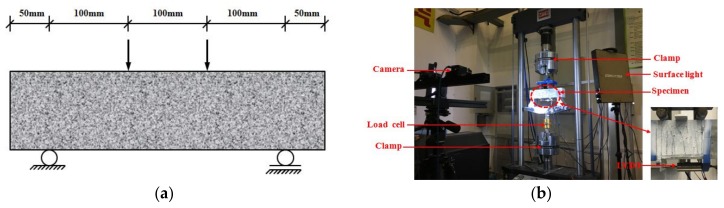
Set-up of the four-point bending test: (**a**) dimension of beam specimens; and (**b**) the VIC-3D set-up.

The test was carried out on a 250 kN MTS fatigue testing system. The static loading test was displacement-controlled at a rate of 1 mm/min. The fatigue test was carried out under load-control at a sinusoidal frequency of 8 Hz. The loading device for the static loading and the fatigue test used a four-point bending procedure (Figure 6). The middle segment was a pure bending section, and the distance between the supports was 300 mm; the loading points were located on the left third of the beam and right third of the beam with a spacing of 100 mm. Three LVDTs were installed to measure the deflections at the supports and the mid-span, respectively. A LVDT was arranged along the axial direction of the specimen in the pure bending section of the beam bottom to measure the COD of the pure bending section. During the test, the lateral crack width of the test beam was measured with a crack observation instrument.

### 2.4. Fatigue Loading Scheme

**Figure 7:**
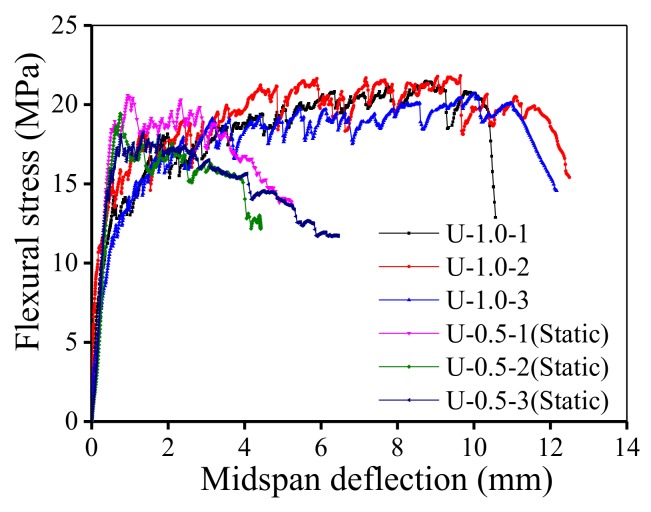
Flexural stress-midspan deflection relationship.

The flexural strength *f*_u_ and the average flexural strength *f*_a_ were calculated from the ultimate load *P*_u_ of the specimen under static loading. The flexural strength *f*_u_ is the peak flexural stress of flexural stress-midspan deflection curve shown in Figure 7. As previously noted, the stress level *S* is defined as the ratio of the maximum fatigue stress σ_max_ to the average flexural strength. The minimum fatigue stress was set to be 20% of the maximum fatigue stress herein. A total of four levels of stress were set, namely, 0.5, 0.65, 0.8, and 0.9, which cover fatigue characteristics from the high-cycle fatigue zone to the low-cycle fatigue zone. It is generally believed that concrete structures will not fail under the condition of fatigue loading if they can undergo up to 2 million cycles of fatigue loading without damage [18]. Therefore, the test was stopped when the specimen failed or the number of loading cycles reached 2 million.

## 3. Results and Discussion

### 3.1. Static Loading Test

**Figure 8:**
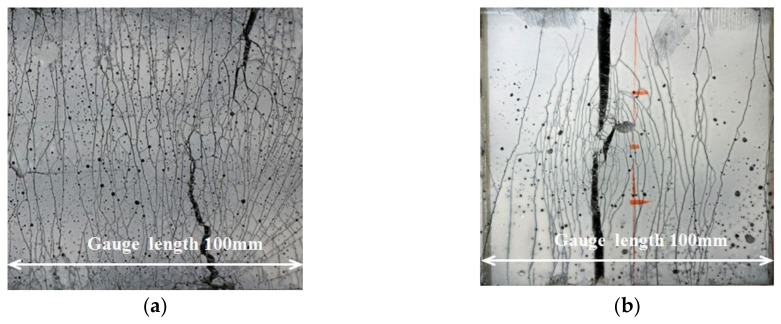
Cracks in the bottom of specimen: (**a**) U-1.0-*n* where *S* = 1.0; and (**b**) U-0.5-*n* (static) where *S* = 0.5.

Figure 7 shows the flexural stress-midspan deflection curves of the four-point bending beams under statically loading. After 2 million fatigue loading cycles at a stress level of 0.5 without failure, the UHP-ECC specimens were statically reloaded to failure; then, their flexural behavior was compared to that of the UHP-ECC specimen, which was directly statically loaded to failure. These two types of specimens were respectively identified as U-0.5-*n* (static) and U-1.0-*n*, where *n* = 1, 2, 3. A suffix of “(static)” was appended to U-0.5-*n* to indicate the specimens were statically reloaded after fatigue loading. The results show that the average ultimate flexural stress of the specimens U-1.0-*n* approached 22 MPa, and the corresponding average mid-span deflection was greater than 10 mm. The average deflection-to-span ratio was greater than 1/30. Although the flexural strength after fatigue loading was reduced to 86% of that under direct static loading and the strain hardening part of the curve was shortened, the UHP-ECC specimens U-0.5-*n* (static) can still have a tensile strain capacity of more than 3%, indicating the UHP-ECC material had excellent ductility. The superior ductile properties of UHP-ECC were further manifested by its cracking characteristics, which agreed with the results in the flexural stress-mid-span deflection curve. Figure 8 shows the crack development at the bottom of the pure bending section of the UHP-ECC specimen. For specimens statically loaded to failure (U-1.0-*n*, where *S* = 1.0), the cracks spread throughout nearly the entire pure bending section with a small crack width and a large crack number; however, a large crack occurred when the specimen finally failed, as shown in Figure 8a. Meanwhile, the specimens that underwent a first fatigue loading and then static loading (U-0.5-*n*, where *S* = 0.5), for only fine cracks, which were distributed around the main cracks. The main crack widths were large, and the number was small, as shown in Figure 8b.

**Figure 9:**
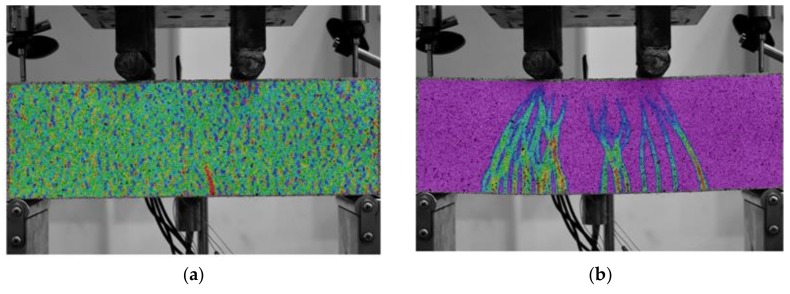

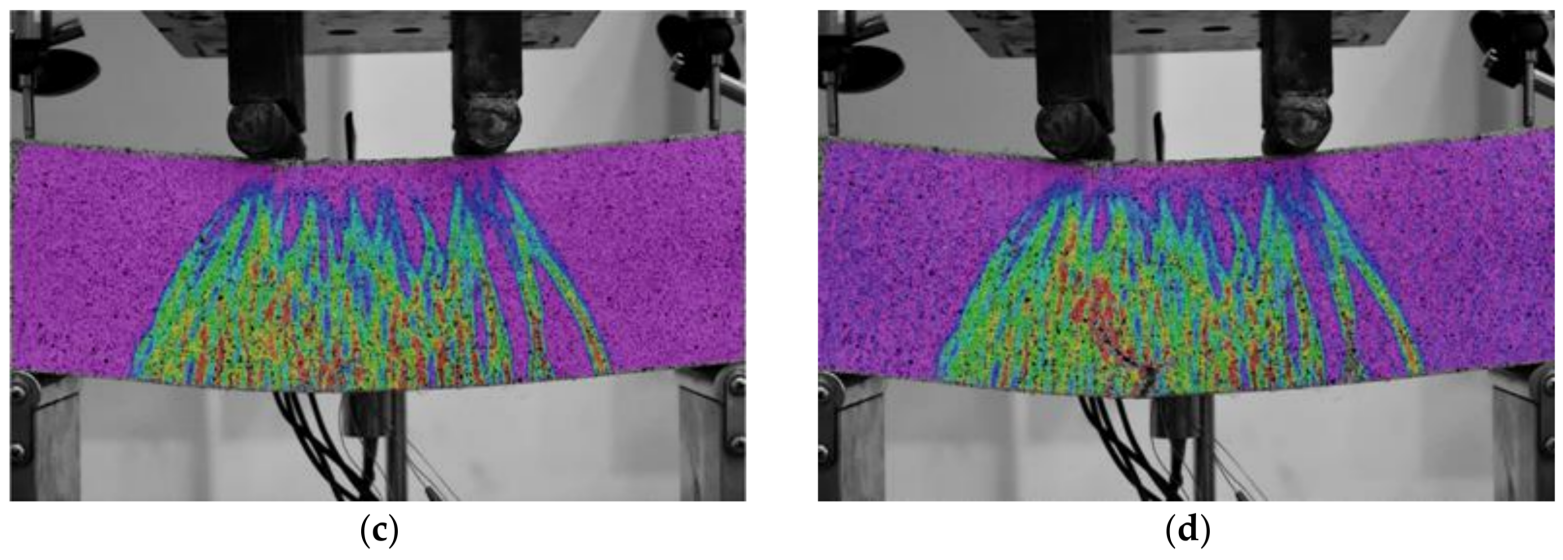
Crack patterns of the flexural beam at different loading stages: (**a**) First crack (40 kN); (**b**) 18th crack (50 kN); (**c**) Peak load (69 kN); and (**d**) 1/30 deflection-to-span ratio (62 kN).

In addition, the crack development process of U-1.0-3 was observed using a DIC during the loading process. The observation results are shown in Figure 9. The first crack appeared when the load reached 58% of ultimate load (40 kN); afterwards, the number of cracks continuously increased, and when the load reached 72% of ultimate load, (50 kN), the number of cracks was 18; when the peak load (69 kN) was reached, multiple cracks were observed in the entire pure bending section, showing an excellent ductility. When the deflection-to-span ratio reached 1/30 (62 kN), the number of cracks tended to be saturated, large cracks (shown in the red circle) appeared locally, and the bearing capacity began to decrease, as shown in Figure 9d.

### 3.2. Fatigue Testing

#### 3.2.1. Failure Modes

**Figure 10:**
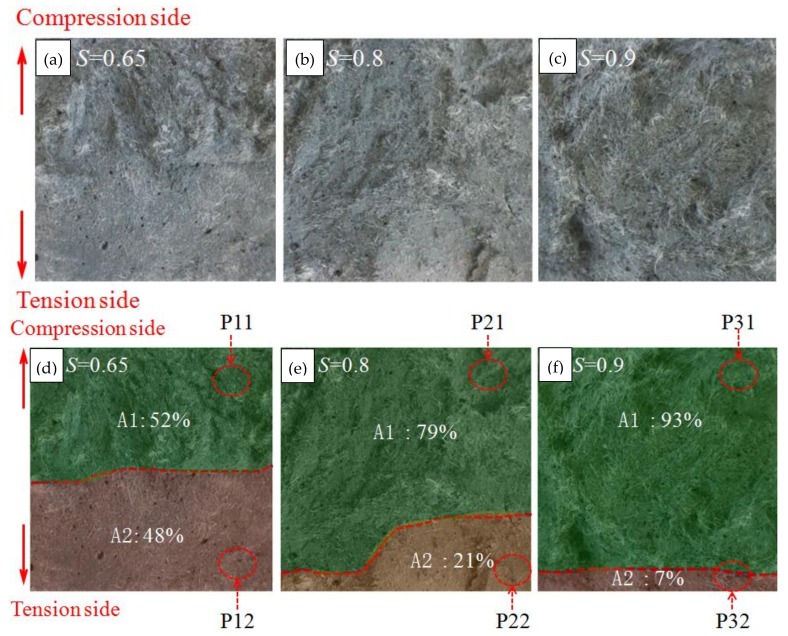
Failure sections of UHP-ECC at different stress levels: (**a**) *S* = 0.65; (**b**) *S* = 0.8; (**c**) *S* = 0.9 before image processing; and (**d**) *S* = 0.65; (**e**) *S* = 0.8; (**f**) *S* = 0.9 after image processing.

When loaded with different fatigue stress levels, the fractured surface of the specimen exhibited different failure modes as shown in Figure 10. Figure 10a–c indicates that the fractured surface could be divided into two parts where two different failure modes of fiber pull-out and fiber rupture were observed. With the increase of the stress levels, the fractured surface had a different ratio of ruptured fibers to pulled fibers. Figure 10d–f further show a plot to quantify the fiber pull-out area and rupture area at the fractured surface of specimens loaded with different stress levels, where the pull-out and rupture areas were labeled as A1 and A2, respectively. The pull-out area was close to the side of the compression zone of the beam where the surface was uneven and the fibers were mainly pulled out. The fiber rupture zone was close to the side of the tension zone where the section was smooth and the fiber was mainly ruptured. As the fatigue stress level increased, the area ratio of A1 to A2 significantly increased; thus, the ratio of pulled fibers to ruptured fibers increased. Hence, it can be concluded that high fatigue stress has a more significant degradation effect on the bond performance between fibers and the matrix than that on the mechanical properties of the fibers; while low fatigue stress mainly damages the fibers.

**Figure 11:**
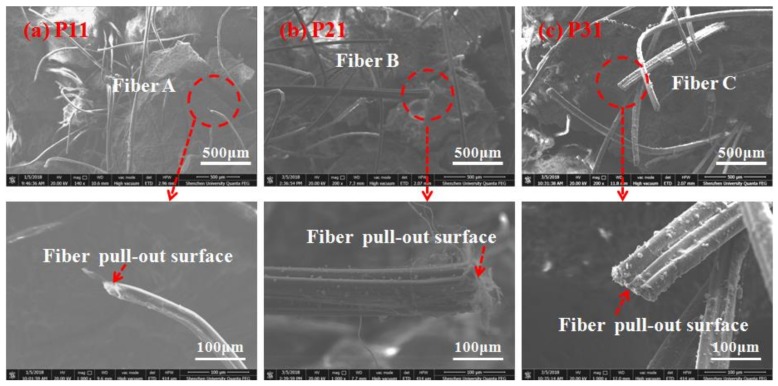
SEM images of the fiber pull-out surface: (**a**) P11; (**b**) P21; (**c**) P31 as marked in Figure 10d–f; respectively.

**Figure 12:**
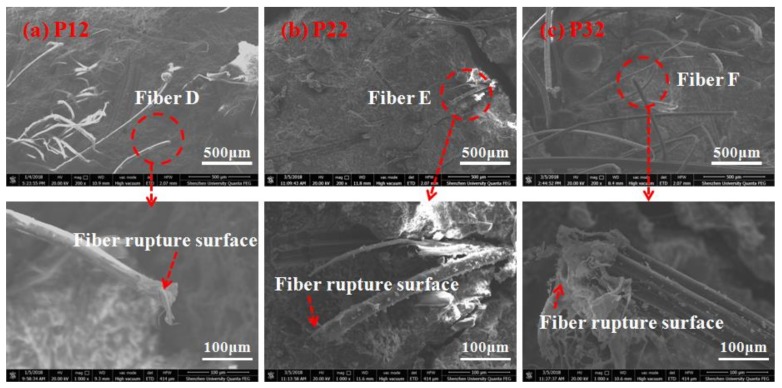
SEM images of fiber rupture surface: (**a**) P12; (**b**) P22; (**c**) P32 as marked in Figure 10d–f; respectively.

Figure 11 and Figure 12 compares the failure morphologies of the fibers in the rupture zone and the pull-out zone. Samples from different regions in the fractured section were analyzed using an environmental scanning electron microscope (ESEM). ESEM was used to capture images of specimens in the rupture zone and pull-out zone, i.e., P11, P21, P31 and P12, P22, P32 in Figure 10d–f. The samples were oven dried at 50 °C for two days. Afterwards, the samples were analyzed by using a multistage vacuum system and a gas secondary electron signal detector. Figure 11 shows the electron microscope images of the fiber pull-out zone, which were mainly composed of pulled out-fibers. The fibers had a severely scratched surface and were gradually changed from a round shape to a flat shape with the increase of the stress level. UHP-ECC specimens under low stress levels mainly underwent elastic deformation, which was insignificant, and it was easy for fibers to maintain the round shape since that is their original state; while under high stress levels, the plastic deformation played a dominant role, which was large during the loading and unloading stages of each cycle; therefore, most fibers were squeezed by the matrix and marked by scratches during the pullout process. Figure 12 shows the electron microscope images of the fiber ruptured zone. The fibers have a round shape that is severely damaged and torn with a noticeable wire drawing phenomenon. Moreover, the length of the free-hanging ruptured fibers from the matrix is significantly shorter than that of the pulled fibers.

#### 3.2.2. Deformation Characteristics

The initial stiffness of the UHP-ECC specimens before and after fatigue loading was also compared. The initial stiffness is 40% of the peak flexural stress serving as a ratio to the corresponding midspan deflection. The results are summarized in Table 4. The average initial stiffness of the specimens under static loading and after fatigue loading was approximately measured to be 130.01 kN/mm and 108.12 kN/mm, respectively. After fatigue loading, the stiffness was degraded—mostly due to the fiber failure mode in the tension zone changing from, mainly, rupture failure under static loading to the pullout failure mode, which further reflects the bond-slip effect between the fiber and the matrix after fatigue loading, as discussed previously.

**Figure 13:**
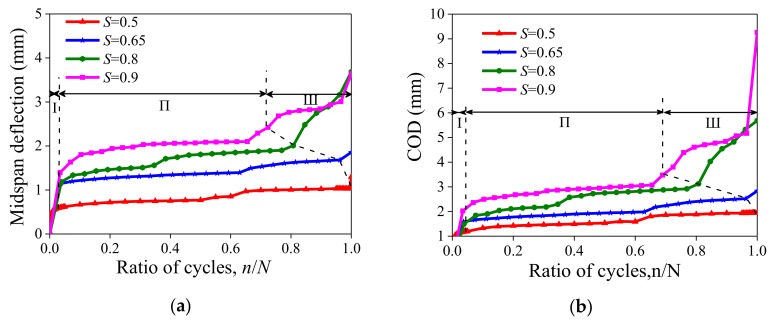
Evolution curve of deflection with cycle loading: (**a**) midspan deflection; and (**b**) crack opening displacement (COD) of bottom.

During the test, besides the midspan deflection, the crack opening displacement (COD) of the pure bending section was also measured with a LVDT installed parallel to the beam’s mid-span section as shown in Figure 6b. Figure 13 shows the evolution curve of mid-span deflection and the COD and ratio of fatigue loading cycles under different stress levels, where n is the number of loading cycles and N is the fatigue life of the specimen. This figure also shows that the midspan deflection and COD of the specimen increased with the increase of stress level. When the stress level is small, e.g., *S* = 0.5, the midspan deflection and the COD undergone two stages, namely rapid deformation growth (Stage I) and deformation stabilization (Stage II). The deformation mainly occurred in Stage I. The deflection in this stage included the elastic displacement of the specimen and deflection caused by the specimen cracking after dynamic disturbance. However, as the stress level increases, e.g., *S* = 0.65, 0.8 and 0.9, the whole deformation process can be divided into three stages: the above-mentioned Stage I and II, plus Stage III, where instability and failure occurred. The deformation occurred mainly in Stage I and Stage III. Stage II is the stable development stage of cracks, and the rate of deformation is positively correlated with the stress level. The UHP-ECC specimens exhibited the characteristics of ductile failure under fatigue loading. In the case of high fatigue stress, the deformation was obvious with many cracks. All specimens under different stress levels experienced a stable Stage II of deformation. The proportion of Stage II in the fatigue life cycle varied with the stress levels. Stage I was mainly characterized by elastic deformation and the rapid expansion of micro-cracks under fatigue loads. Especially in the case of small stress (e.g., *S* of 0.5 and 0.65), the elastic deformation was the main deformation. When the specimen entered the second stage, new cracks were generated with the continuous application of fatigue loads, and the deformation of the specimen was mainly caused by the propagation of the original cracks and the generation of new cracks. In the third stage, the main crack appeared. However, due to the presence of fibers, the specimen did not fail immediately. Instead, the fibers at the fractured surface were continuously broken or pulled out. The rapid increase of the main crack width (see Figure 8) accelerated the deformation of the specimen and weakened the bending strength of the specimen, causing a sudden break of the specimen. The fatigue life mainly depended on the length of Stage II and the magnitude of deformation, which were closely related to the number and density of the cracks.

#### 3.2.3. Crack Observation

**Figure 14:**
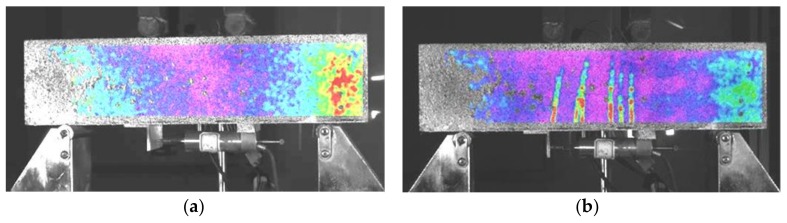

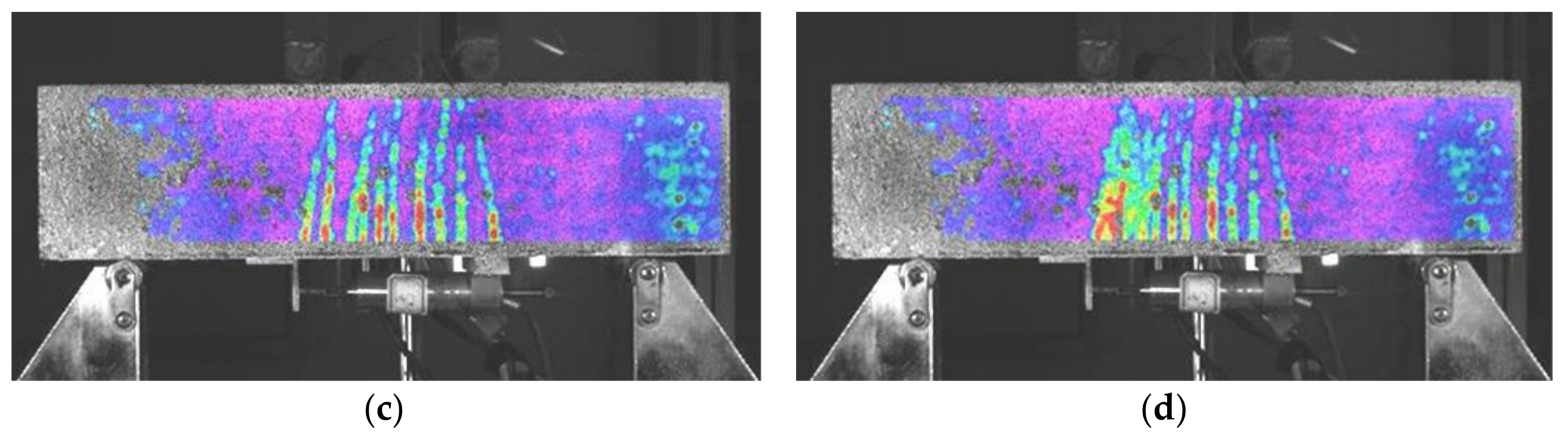
Crack patterns of flexural beam at *S* = 0.8 by DIC: (**a**) no crack (29 cycles); (**b**) fifth crack (30 cycles); (**c**) ninth crack (200 cycles); and (**d**) main crack (20,000 cycles).

The crack development under fatigue cyclic loading where *S* = 0.8 was observed by the digital image technique DIC. The results are shown in Figure 14. No cracks (Figure 14a) occurred until the number of load cycles reached 30 (Figure 14b); then, the first batch of cracks appeared. When the number of load cycles reached 200 (Figure 14c), the number of cracks increased to 9, and the cracks no longer increased in size. After 200,000 load cycles, large cracks (shown in the red circle in Figure 14d) appeared locally, and the specimen was about to break. Similar to the cracks under static loading, as shown in Figure 9, the cracks occurred mainly in the pure bending section and its vicinity, but the number of cracks and the ductility were significantly reduced.

**Figure 15:**
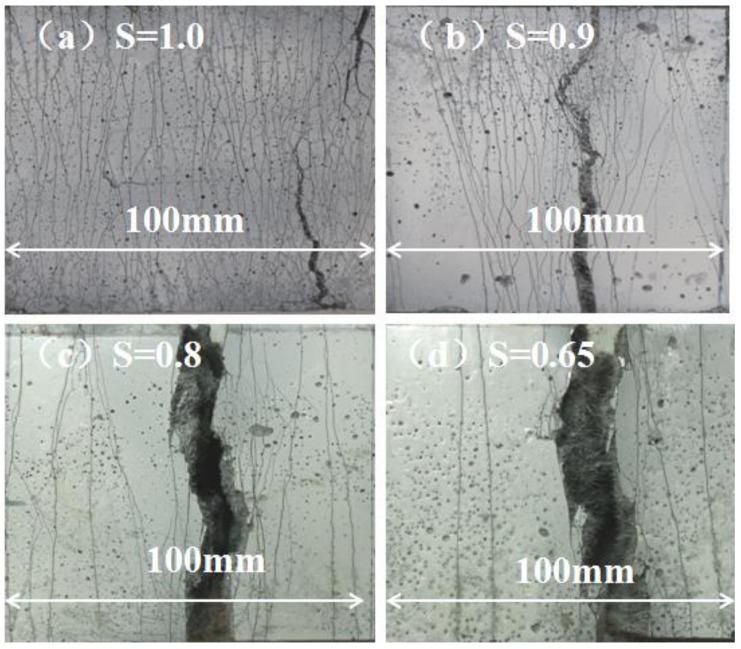
Cracks in the bottom of specimen: (**a**) *S* = 1.0; (**b**) S = 0.9; (**c**) *S* = 0.8; (**d**) *S* = 0.65.

**Figure 16:**
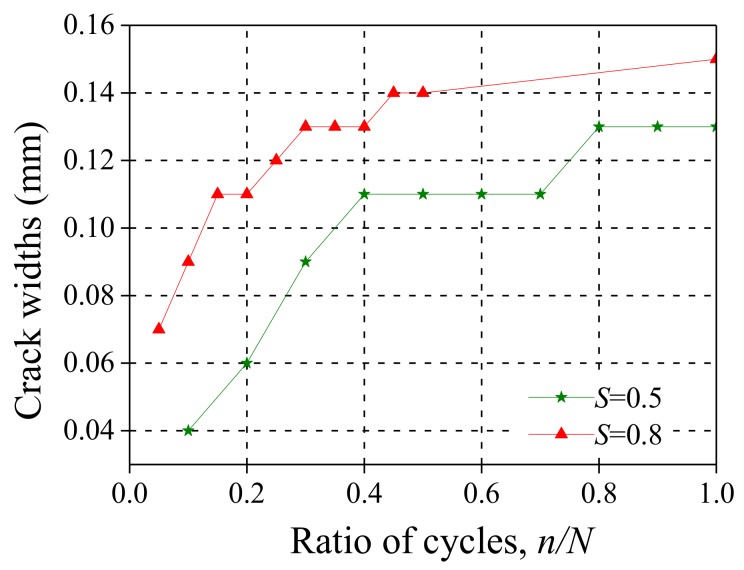
Crack widths in the bottom of specimen at *S* = 0.5 and *S* = 0.8.

Figure 15 shows the cracks in the pure bending section at the bottom of the specimen after different stress levels of fatigue loading. Table 5 shows the average number of cracks at each stress level. As the stress level increases, the number of cracks gradually increased. Figure 16 further shows the crack width development under fatigue loading. It is seen that the crack width increases as the fatigue cycles increase; thus the crack width at *S* = 0.8 was significantly larger than that at *S* = 0.5, resulting in a significant decrease of the fatigue life as shown in Table 6.

#### 3.2.4. Energy Dissipation

**Figure 17:**
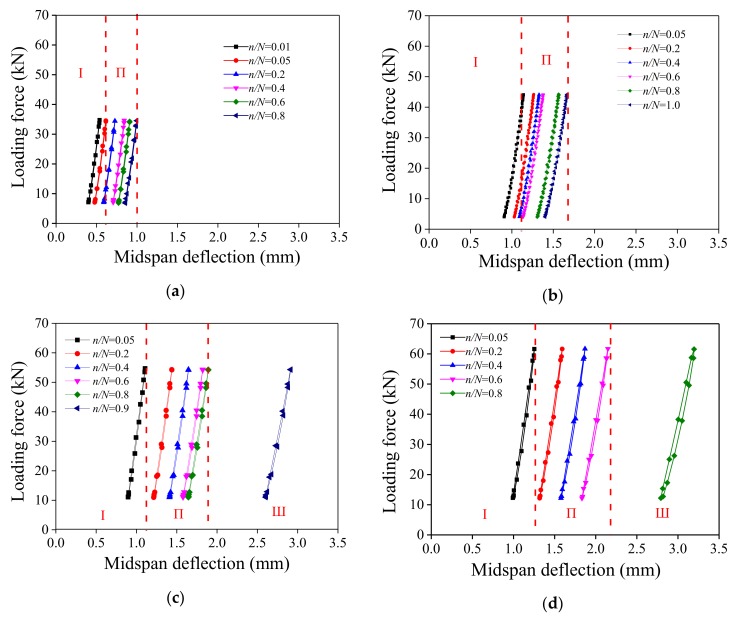
Hysteretic curves of specimens in given loading cycles at different stress levels or deformation stages (I = rapid deformation; II = deformation stabilization; and III = instability and failure): (**a**) *S* = 0.5; (**b**) *S* = 0.65; (**c**) *S* = 0.8; (**d**) *S* = 0.9.

The hysteresis curves of the UHP-ECC at different fatigue stress levels are shown in Figure 17 where the aforementioned three deformation stages are also identified as Stage I (rapid deformation), Stage II (deformation stabilization), and Stage III (instability and failure), depending on the fatigue stress. With the increase of the fatigue stress level, the mid-span deflection was mainly distributed in a region ranging between 0.25 and 1.0 mm at *S* = 0.5 as well as between 1.0 and 3.0 mm at *S* = 0.9, respectively. Most of the load cycles took place in Stage II, and the load-midspan deflection curves exhibit a linear characteristic.

**Figure 18:**
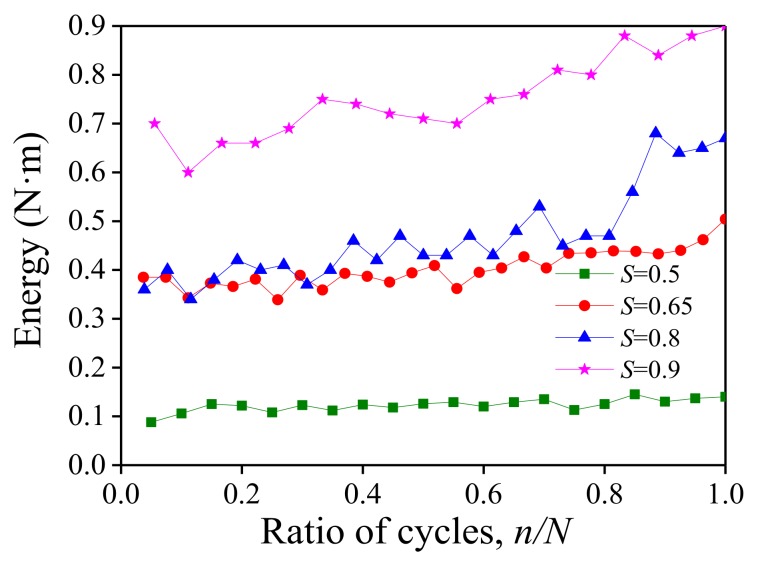
Evolution curve of energy with cycle loading at different stress levels.

Moreover, with the increase of the fatigue loading cycle especially when the deformation of the UHP-ECC entered Stage III at high stress level, the single hysteresis loop became fuller, and more energy was dissipated. The energy dissipation in each hysteresis curve during each cycle was calculated. The curves of the dissipated energy show how the cycle ratio under different stress levels were obtained, as shown in Figure 18, where the dissipated energy in a single cycle increased slowly with the cycle ratio over the entire fatigue life cycle, and the rate also increased slightly with the stress levels.

## 4. Fatigue Life Models

**Figure 19:**
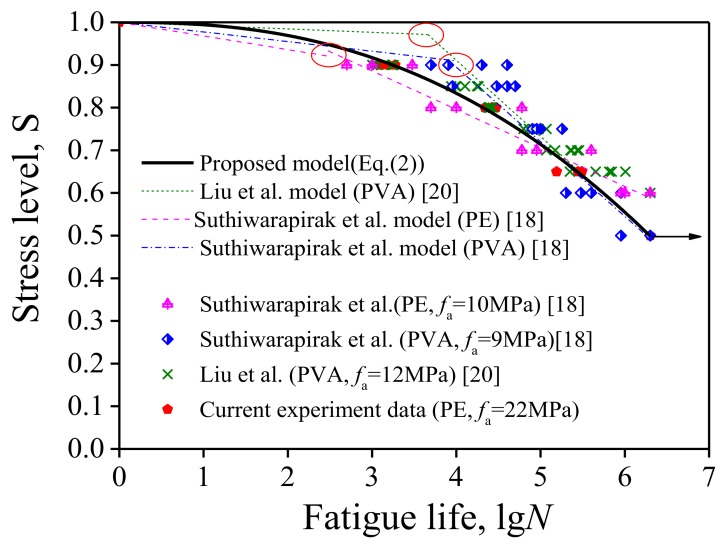
Stress level-fatigue life curve.

**Figure 20:**
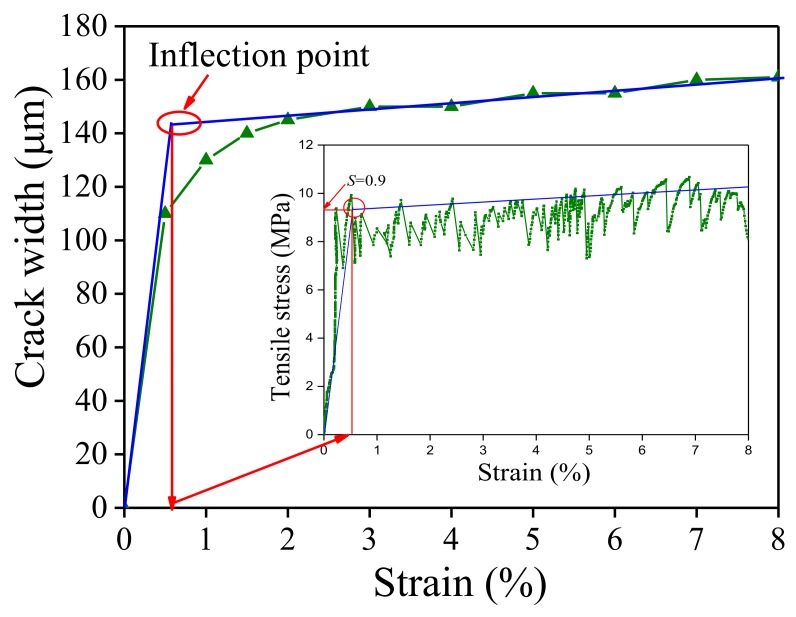
Relation of inflection point between critical crack width and stress level.

Many scholars have conducted in-depth studies modeling ECCs using single- and double-logarithms [16,17,18,19,20], as summarized in Table 7, where the relation between the fatigue stress level S and failure fatigue cycles N, i.e., the S-N equation, was used to assess the fatigue life of ECCs. Figure 19 illustrates the typical bilinear S-N curves. There exists an inflection point in all the S-N curves. The number of cycles corresponding to the inflection point of all the existing models ranges between 10^2^ and 10^4^, and the corresponding stress level is around 0.9. Although different models proposed different inflection points, the present test result indicates that the stress level of the inflection point was in good agreement with the stress corresponding to the inflection point of the crack width development curve with the strain as shown in Figure 20, indicating that the occurrence of this inflection point was related to a critical crack width. Since very limited data were available to accurately quantify the inflection point or, more importantly, the three models (as defined in Table 7), and the two stages are not continuous at their inflection point (marked with red circle in Figure 19). Thus, a continuous nonlinear equation is proposed as follows to characterize the relationship between the stress level (*S*) and fatigue life (*N*) to omit the inflection point:

(1) $$S=1-a{\left(\mathrm{lg}N\right)}^{b}$$

where *S* is the stress level, *N* is the fatigue life, while a and b are the parameters determined by the fatigue test.

Table 6 summarizes test results of the fatigue life and deflection at various stress levels. Through a regression analysis, the fatigue equation of UHP-ECC is obtained:

(2) $$S=1-5.75\times 10^{-3}{\left(\mathrm{lg}N\right)}^{2.43}$$

The *S*-*N* curve obtained by nonlinear fitting is shown in Figure 19 where the existing bilinear fatigue life models were evaluated and compared. Notably, the arrow in the figure means that the specimen was not broken after 2 million fatigue cycles, and the test was, thus, stopped.

As can be seen from Figure 19, the fatigue performance of the high-strength ECC is not inferior to that of ordinary ECC. Furthermore, PVA-ECC exhibited a higher fatigue life than PE-ECC at high stress levels, but had a slightly poorer fatigue performance than PE-ECC under low stress levels. This may be attributed to the higher bond strength, but lower fatigue performance, of PVA fibers when compared with PE fibers [29]. As previously mentioned, most of the fibers were pulled out from the matrix at high fatigue stress but ruptured at low fatigue stress; therefore, the better bond performance of PVA fibers will lead to superior crack width control capability for PVA-ECC [32]. Thus, the fatigue life for PVA-ECC improves when subjected to high fatigue stress. On the other hand, at low fatigue stress, both the failures of PVA-ECC and PE-ECC are dominated by fiber rupture; thus, a better fatigue performance of PE fibers will result in a higher fatigue life for PE-ECC when compared with PVA-ECC.

## 5. Conclusions

In this paper, the UHP-ECC specimens were subjected to fatigue tests under different fatigue loading stresses. The fatigue stress was set at four different fatigue stress levels: 0.5, 0.65, 0.8, and 0.9. Final measurements and analyses were discussed pertaining to the maximum number of fatigue cycles when UHP-ECC fails at each stress level as well as the corresponding deformation characteristics of the specimens, which included crack widths an hysteretic energy dissipation. The following conclusions were obtained:

(1) The UHP-ECC specimens exhibited a ductile failure after fatigue loading and the high strength matrix of the UHP-ECC specimen did not affect its fatigue life. The final fatigue failure is mainly due to the accumulated damage of the fiber and its bond performance between the cement matrices. The fractured surface of the UHP-ECC composites were filled with pulled-out fiber failure and fiber rupture failure. With the increase of the fatigue stress, the ratio of the fiber pulled-out area to the fiber ruptured area on the fractured surface increases significantly. The fibers with pull-out failure had a severely scratched surface and were gradually changed from a round shape to a flat shape with the increase of the stress level; and the fibers with rupture failure had a round shape and were severely damaged and torn with a noticeable wire drawing phenomenon.
(2) The midspan deflection of the UHP-ECC increased with the increase of the fatigue stress level and its development underwent two or three stages, namely rapid deformation growth (Stage I), deformation stabilization (Stage II), and instability and failure (Stage III), depending on the fatigue stress. The deformation of Stage II was mainly caused by the propagation of the original cracks and the generation of new cracks. The fatigue life mainly depended on the length of Stage II and the magnitude of deformation, which were closely related to the number and density of the cracks.
(3) Due to the higher bond strength but lower fatigue performance of PVA fibers when compared with PE fibers, PVA-ECC exhibited a higher fatigue life than PE-ECC under high stress levels, but had a slightly poorer fatigue performance than PE-ECC under low stress levels.
(4) Existing studies widely use a developmental stage model with an inflection point to predict fatigue the fatigue life of ECC, i.e., the relationship between the stress level (S) and fatigue cycle (N), also referred to as the, S-N curve. However, very limited data has been available to accurately quantify the inflection point and all of the existing developmental stage models are not continuous at their inflection point. Thus, a continuous nonlinear equation is proposed to characterize the S-N curve of the UHP-ECC. The results indicate the proposed fatigue life model can produce a reliable prediction.

## Figures and Tables

**Table 1:** Composition of UHP-ECC under investigation (kg/m^3^).

OPC ^1^	LP ^2^	GGBS ^3^	SF ^4^	QS ^5^	HRWR ^6^	PE	Water
700	100	750	150	500	30	20	230

^1^ Ordinary Portland cement. ^2^ Limestone powder. ^3^ Ground granulated blast furnace slag. ^4^ Silica fume. ^5^ Quartz sand. ^6^ Polycarboxylate-based liquid high range water reducer.

**Table 2:** Physical, chemical, and mechanical properties of cement, silica fume and slag.

Components	Chemical Composition (%)				Properties of Cement	
	OPC	SF	GGBS	LP		
SiO_2_	20.10	92.26	39.66	0.07	Specific gravity (g/cm^3^)	3.13
CaO	62.92	0.49	34.20	56.90	Blaine fineness (m^2^/kg)	380
Al_2_O_3_	5.62	0.89	12.94	0.00	Initial setting times (min)	130
Fe_2_O_3_	2.17	1.97	1.58	0.02	Final setting times (min)	210
MgO	1.14	0.96	6.94	0.13	Volume expansion (mm)	1.00
Na_2_O	0.30	0.42	0.20	0.07	Compressive strength	53.2 7-day
K_2_O	0.85	1.31	1.44	0.00	(MPa)	61.9 28-day
SO_3_	2.92	0.33	0.72	0.05		
L.O.I.	3.84	<6.00	1.20	42.73		

**Table 3:** Properties of PE fiber.

Diameter Ratio (μm)	Fiber Aspect Ratio	Volume Percentage (%)	Strength (GPa)	Elastic Modulus (GPa)	Rupture Elongation (%)	Density (kg/m^3^)
25	480	2	2.9	116	2.42	970

**Table 4:** Experimental stiffness results.

Specimen *	40% Ultimate Flexural Load (kN)	Related Midspan Deflection (mm)	Initial Stiffness *B*_0_ (kN/mm)	Average Initial Stiffness (kN/mm)
U-1.0-1	27.21	0.21	129.57	130.01
U-1.0-2	29.09	0.20	145.45	
U-1.0-3	27.60	0.24	115.00	
U-0.5-1 (static)	26.00	0.24	108.33	108.12
U-0.5-2 (static)	26.53	0.27	98.26	
U-0.5-3 (static)	24.73	0.21	117.76	

* U-0.5-1 (static) denotes the specimens were first applied a fatigue load and then static load up to failure.

**Table 5:** Average number of flexural cracks at each stress level.

Stress Level (*S*)	1 (Static)	0.9	0.8	0.65	0.5 (before Static)	0.5 (after Static)
crack number	45	30	20	6	5	25

**Table 6:** Results of flexural fatigue test at each stress level.

Stress Level (*S*)	Specimen	Fatigue Life (*N*)	Max Midspan Deflection (mm)	Max Deflection of Bottom (mm)
0.5	U-0.5-1	(2,000,000) ^1^	0.99	1.61
	U-0.5-2	(2,000,000) ^1^	1.03	1.94
	U-0.5-3	(2,000,000) ^1^	0.98	1.96
0.65	U-0.65-1	308,691	1.76	2.64
	U-0.65-1	155,075	1.41	2.76
	U-0.65-1	277,386	1.86	2.83
0.8	U-0.8-1	29,565	3.64	3.97
	U-0.8-2	26,291	3.68	3.13
	U-0.8-3	21,852	3.02	3.77
0.9	U-0.9-1	1569	4.81	5.21
	U-0.9-2	1876	4.85	4.92
	U-0.9-3	1271	4.94	4.37

^1^ The data in the parentheses indicates the specimen did not fail after 2 million cycles and the test was, thus, stopped.

**Table 7:** Model of fatigue life in the existing literature.

Literature	Year	Model
Liu et al. (PVA) [20]	2017	$\{\begin{array}{c} \mathrm{lg}S=0.0011-0.0165\mathrm{lg}N,\;\;\;\;1<N<10^{4}\\ \mathrm{lg}S=0.1974-0.0654\mathrm{lg}N,\;\;\;\;\;\;\;\;\;\;\;\;\;N\geq 10^{4} \end{array}$
Suthiwarapirak et al. (PVA) [18]	2004	$\{\begin{array}{c} S=1.000-0.0226\mathrm{lg}N,\;\;\;\;\;\;\;\;\;\;\;\;1\leq N<1\times 10^{4}\\ S=1.595-0.1750\mathrm{lg}N,\;1\times 10^{4}\leq N\leq 2\times 10^{6} \end{array}$
Suthiwarapirak et al. (PE) [18]	2004	$\{\begin{array}{c} S=1.000-0.0320\mathrm{lg}N,\;\;\;\;\;\;\;\;\;\;\;\;\;\;\;\;\;\;1<N<3\times 10^{2}\\ S=1.157-0.0903\mathrm{lg}N,3\times 10^{2}\leq N<2\times 10^{6} \end{array}$
